# Colonoscopic withdrawal time and adenoma detection in the right colon

**DOI:** 10.1097/MD.0000000000012113

**Published:** 2018-08-21

**Authors:** Gee Young Yun, Hyuk Soo Eun, Ju Seok Kim, Jong Seok Joo, Sun Hyung Kang, Hee Seok Moon, Eaum Seok Lee, Seok Hyun Kim, Jae Kyu Sung, Byung Seok Lee, Hyun Yong Jeong

**Affiliations:** Division of Gastroenterology, Department of Internal Medicine, Chungnam National University School of Medicine, Chungnam National University Hospital, Daejeon, Republic of Korea.

**Keywords:** adenoma detection rate, cap-assisted colonoscopy, colonoscopic withdrawal technique, colonoscopic withdrawal time, interval colorectal cancer, right colon

## Abstract

Shorter colonoscopic withdrawal time (CWT) has been associated with lower adenoma detection rate (ADR), which can increase the risk of interval colorectal cancer (ICC) that commonly arises in the right colon (RC). Therefore, a better ADR in the RC could decrease the incidence of ICC. We analyzed the relationship between CWT and ADR in the RC and entire colon.

We retrospectively reviewed the patients who had undergone screening colonoscopy at Chungnam National University Hospital between March 2015 and February 2016. We enrolled 5370 patients in whom colonoscopies were performed by 7 gastroenterologists. We categorized patients into 4 groups in the RC and 6 groups in the entire colon by CWT. Multivariable analysis was used for detection of adenoma in the RC and entire colon.

In the RC, the odds ratio (OR) of CWT longer than 3 minutes was 3.70, compared to CWT of <2 minutes [3.06–4.85, 95% confidence interval (CI), *P* < .001]. In the entire colon, the OR of CWT between 9 to 10 minutes and longer than 10 minutes was 3.34 [2.61–4.27, 95% CI, *P* < .001] and 3.49 [2.80–4.33, 95% CI, *P* < .001] compared to CWT of <6 minutes.

Based on our result, we suggest that the optimum CWT in the RC should exceed 3 minutes, and considering the “ceiling effect,” the optimum CWT in the entire colon should exceed 9 minutes.

## 1 Introduction

Colorectal cancer is an important disease, one of the leading causes of cancer death, across ages and in all countries. There have been many advances in preventing colorectal cancer. Screening by colonoscopy is useful for detecting colorectal adenomas and helps in reducing colorectal cancer. However, the current challenging issue includes how to reduce interval colorectal cancer (ICC), especially for the right colon (RC), which colonoscopy is less effective.^[1]^

Colonoscopic withdrawal time (CWT) and adenoma detection rate (ADR) are widely used quality indicators for screening colonoscopy.^[2]^ Barclay et al^[3]^ suggested that CWT should be longer than 6 minutes for higher ADR, whereas Simmons et al^[4]^ suggested that it should be longer than 7 minutes for higher polyp detection rate. Lee et al^[5]^ suggested that CWT should be longer than 6 minutes, and that around 10 minutes is necessary for higher ADR. In addition, shorter CWT has been associated with lower ADR, which can increase the risk of ICC.^[6]^ ICCs often arise in the RC.^[7]^ Therefore, improvement of ADR in the RC may decrease the incidence of the ICCs. One recent study published in Taiwan found that shorter withdrawal time in the ascending colon was associated with the development of ICC.^[8]^

There are many methods to increase ADR in the RC, such as retroflexion^[9]^, retroscope,^[10]^ repeating forward view examinations,^[11]^ changing positions of the patient,^[12]^ and cap-assisted colonoscopy (CAC).^[13]^ We hypothesized that all of the above methods may be associated with increasing the CWT of the RC, and thus eventually increasing the ADR of the RC. Several previous studies analyzed CWT and ADR with the whole colon and not particularly in the RC. The aim of our study was to determine if there was a relationship between CWT and ADR in the RC. If there was, we tried to find the optimum CWT in the RC. The relationship between the CWT and ADR in the complete colon and other modifiable factors influencing ADR were also analyzed.

## 2 Materials and methods

### 2.1 Study population

The medical records of patients who underwent screening colonoscopy at Chungnam National University Hospital (CNUH) between March 2015 and February 2016 were analyzed retrospectively. This study included asymptomatic patients who underwent average-risk screening colonoscopy. Seven gastroenterologists performed the colonoscopy using the CV-260L colonoscope (Olympus, Tokyo, Japan). Seven gastroenterologists are professors with a PhD in CNUH gastroenterology department. Gastroenterologists are all veterans with >5 years of experience and perform >500 colonoscopies per year. Colonoscopy performed by a doctor with <1000 colonoscopy experiences was excluded from this study. Other exclusion criteria included the following: patients who had been referred for polypectomy, poor bowel preparation (based on Aronchick scale^[14]^), inability of endoscopist to reach the cecum, familial polyposis, inflammatory bowel disease, gastrointestinal bleeding, prior colon resection and those who cannot calculate the withdrawal time. This study was approved by the institutional review board of CNUH (IRB Number: 2017-03-064).

### 2.2 Data collection and definition

**Figure 1:**
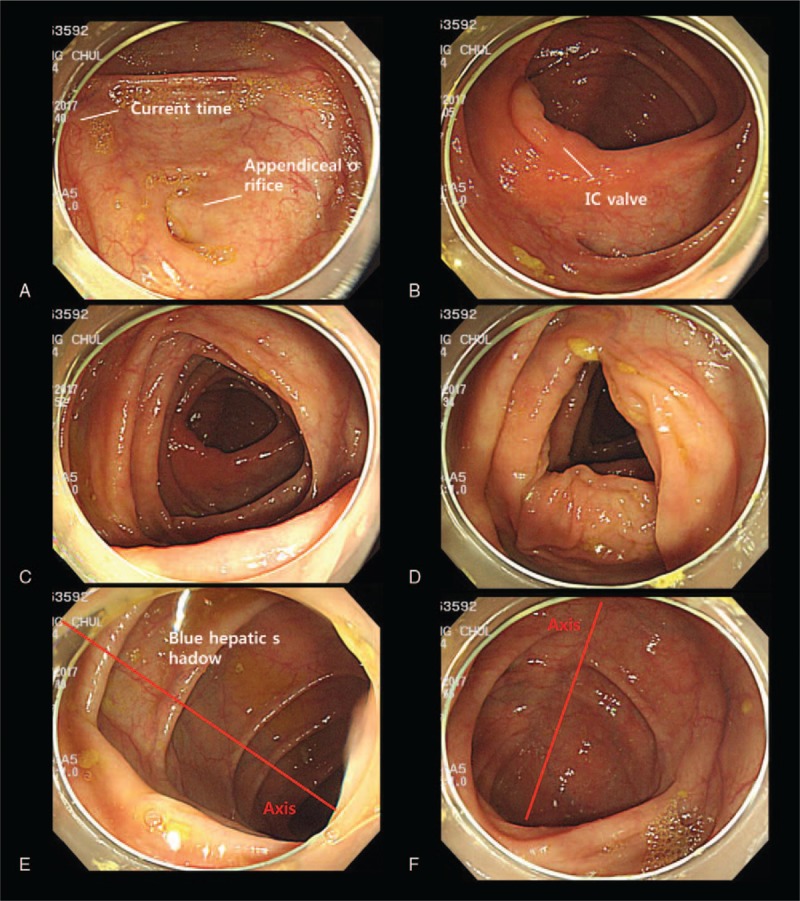
The above images are exported from a picture-archiving communication system (PACS). The current time and patient information are displayed on the left side of the monitor. A, The appendiceal orifice indicates that this area is a cecum. B, The IC valve usually opens to the left, that is, 9 o’clock. C, The IC valve is still visible, indicating that the site is a proximal ascending colon. D, The middle part of the ascending colon. E, Blue hepatic shadow and consecutive colon wrinkles indicate that this area is hepatic flexure. The axis usually starts at 4 o’clock and ends at 10 o’clock. F, The axis has changed and the blue hepatic shadow disappeared. These indicate that this area is a proximal transverse colon.

All clinical and procedural characteristics were confirmed by reviewing medical records and a picture-archiving communication system (PACS). RC was defined as cecum to hepatic flexure, as mentioned in the literature.^[9,11,15–19]^ CWT was calculated by PACS, in which the endoscopists captured the colonoscopic image with the present time simultaneously. We calculated the CWT by subtracting the time between cecum to hepatic flexure for the RC CWT and cecum to anus for total colon (TC) CWT. Hepatic flexure was usually easily identifiable by PACS, but when it was difficult to identify the hepatic flexure because there was no captured image of hepatic flexure, we used the image just before the proximal transverse colon image to calculate the withdrawal time of the RC (Fig. 1). When a polyp or adenoma was discovered, it was removed by snare or forceps. In agreement with the literature, we subtracted all of the polyp -or adenoma-removing time by calculating via PACS.^[10,13,20–23]^

The number, size, site of the polyp or adenoma, sedation state, and cecal intubation state were described in the medical record. And the bowel preparation quality was based on Aronchick Scale.^[14]^ If the endoscopist used the transparent cap for colonoscopy or retroflexed the colonoscope in the RC, we recorded it by reviewing PACS. The final confirmation of the histology of adenoma was based on the pathologists’ report.

ADR is defined as the number of patients who have been discovered to have one or more adenoma during the colonoscopy divided by the total number of colonoscopies. Advanced adenoma is defined as a size >1 cm, villous or tubulovillous adenoma, or high-grade dysplasia, as reported by pathologists.

### 2.3 Statistical analysis

The study data were analyzed using commercial software (SPSS version 22.0, IBM Co, Chicago, IL). Continuous data were presented as mean ± standard deviation. Categorical variables were described with numbers and percentages, unless otherwise specified. A Student *t* test was performed for statistical comparison between the continuous variables, whereas a chi-square test was performed for the categorical data. Binary logistic regression analysis was performed for the detection of adenomas and advanced adenomas in the RC and the TC; all parameters with a *P* value <.1 on univariable analysis were included. Based on CWT, we classified the patients into 4 groups in the RC and 6 groups in the TC to extract the optimum CWT in the RC and the TC. In addition, we used the Spearman rank-correlation coefficient to measure the relationship between the mean CWT which no polyps have been removed and ADR of the gastroenterologists. Two-sided *P* values of <.05 were considered to indicate statistically significant differences.

## 3 Results

We analyzed a total of 6462 patients who underwent average-risk screening colonoscopy from March 2015 to February 2016. There were 663 patients who were referred for polypectomy, and it was the most common reason for exclusion. 139 colonoscopies were excluded due to inadequate bowel preparation and 55 colonoscopies have not reached the cecum. One hundred one patients were excluded due to prior colon resection and 67 patients were excluded due to inflammatory bowel disease. Sixty-seven colonoscopies were unavailable to calculate the withdrawal time. Finally a total of 5370 patients were included in the study and 1092 were excluded. The mean age was 60.7 ± 11.2 years, and 51.7% were of the male sex. The baseline characteristics are summarized in (Table 1).

**Table 1:**
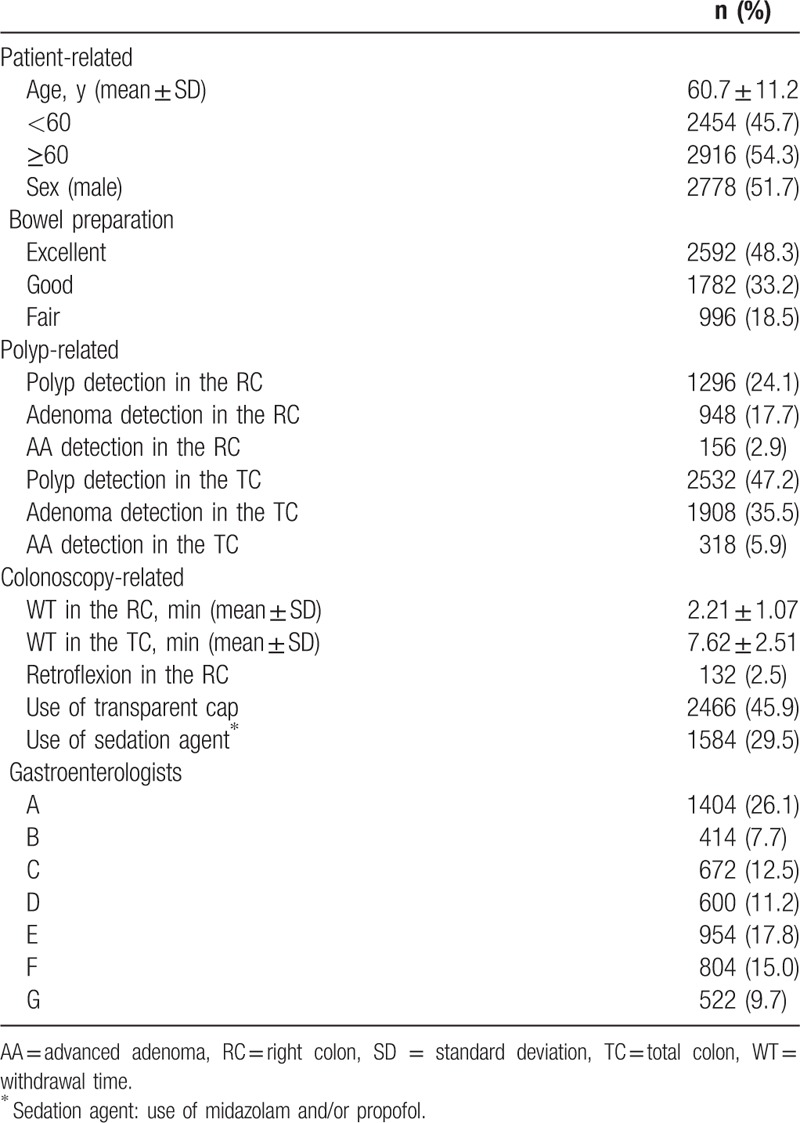
Baseline characteristics (n = 5370).

Gastroenterologists’ ADR in the TC varied from 27.6% to 50.9%, and all of them exceeded 25%, satisfying good performance of the colonoscopy quality indicator.^[24,25]^ Gastroenterologists’ mean CWT in the TC varied from 7 to 10 minutes, and was longer than 6 minutes, satisfying good performance of the colonoscopy quality indicator.^[2,24,25]^

### 3.1 Factors associated with adenoma and advanced adenoma detection in the right colon

Associations between patient-related and colonoscopy-related factors and the detection of adenomas and advanced adenomas in the RC are summarized in Table 2. Significantly more adenomas were detected in elderly patients and men. Good bowel preparation was associated with better adenoma detection in the univariable analysis; when we adjusted for other cofactors, this effect was no longer observed. Of the colonoscopy-related factors, withdrawal time [odds ratio (OR) 1.89, 95% confidence interval (CI) 1.71–2.08, *P* < .001) was significantly associated with better adenoma detection. And adenoma detection differed significantly among gastroenterologists. Advanced adenoma detection was found to be associated with fair bowel preparation in the univariable analysis. This factor was no longer significant when we adjusted for other cofactors. In the multivariable analysis, age, male sex, withdrawal time (OR 1.72, 95% CI 1.56–1.91, *P* < .001) and gastroenterologists were significantly associated with advanced adenoma detection.

**Table 2:**
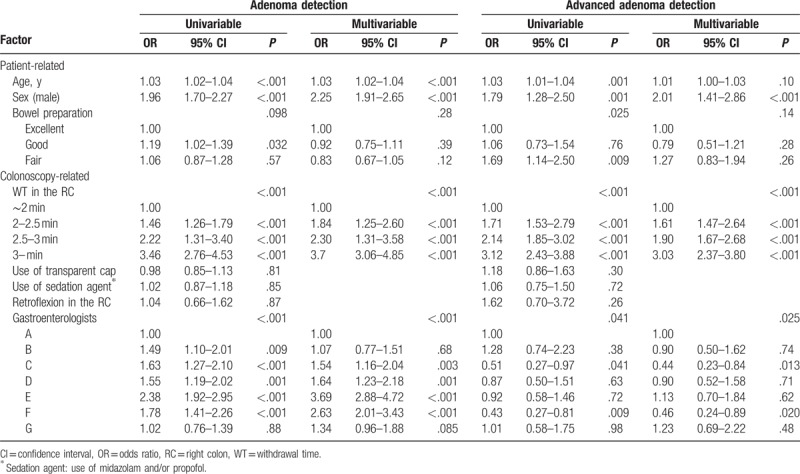
Factors associated with the detection of adenomas and advanced adenomas in the right colon.

### 3.2 Factors associated with adenoma and advanced adenoma detection in the total colon

Associations between patient-related and colonoscopy-related factors and the detection of adenomas and advanced adenomas in the TC are summarized in Table 3. Significantly more adenomas were detected in elderly patients and men. Good bowel preparation was associated with better adenoma detection in the univariable analysis; when we adjusted for other cofactors, this effect was no longer observed. Of the colonoscopy-related factors, withdrawal time (OR 1.25, 95% CI 1.22–1.28, *P* < .001) was significantly associated with better adenoma detection. Using transparent cap was significantly associated with lower adenoma detection. And adenoma detection differed significantly among gastroenterologists. Better advanced adenoma detection was found to be associated with age, male sex, withdrawal time (OR 1.17, 95% CI 1.12–1.22, *P* < .001), and usage of transparent cap in the multivariable analysis. And advanced adenoma detection differed significantly among gastroenterologists.

**Table 3:**
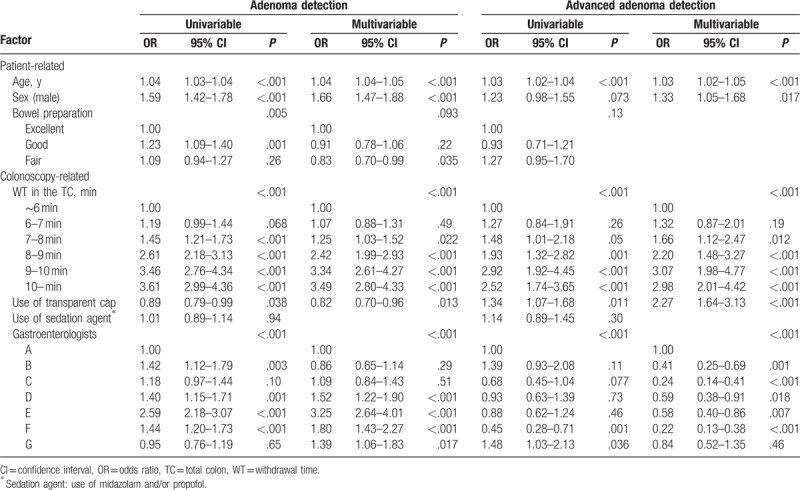
Factors associated with the detection of adenomas and advanced adenomas in the total colon.

## 4 Discussion

In this study, we attempted to find the optimum CWT, particularly in the RC. There are several studies suggesting optimum CWT in the entire colon, and it is recommended that CWT should exceed at least 6 to 7 minutes.^[3,5,6,26]^ In addition, Lee et al^[5]^ suggested that CWT longer than 10 minutes may have no additional benefit in detecting adenomas in colon, demonstrating the ceiling effect.

To our knowledge, this is the first study to report the correlation of the CWT in the RC with the adenoma detection. Lee et al^[5]^ suggested that CWT longer than six minutes, and around 10 minutes, has additional adenoma detection in the RC. However, they did not directly analyze CWT of the RC. Jover et al^[27]^ also suggested that CWT longer than 8 minutes had the best outcome for ADR in both proximal and distal colon, but, they also did not directly analyze CWT in the RC. In our study, we focused on the RC, and calculated its CWT and adenoma detection. Longer WT in the TC does not guarantee the longer WT in the RC. Someone may withdraw faster or slower in the RC for some reasons. Previous studies must have neglected this, so we should carefully withdraw in the RC and the rest of the colon, altogether.

We classified the patients into 4 groups in the RC by CWT. As shown in (Table 2), the OR for the adenoma and advanced adenoma detection of the CWT group of longer than 3 minutes were much higher than that of the lesser CWT groups. Based on our results, the optimum CWT in the RC should exceed 3 minutes. In the TC we classified the patients into 6 groups by CWT. As shown in Table 3, the OR for the adenoma and advanced adenoma detection of the CWT group of longer than 9 minutes were much higher than that of the lesser CWT groups. And there was no significant difference between the 9- to 10-minute group and 10-minute group. This may be explained by the “ceiling effect” mentioned by the previous study.^[5]^ According to these results, the optimum CWT in the entire colon should exceed 9 minutes and it is concordant with the previous studies.^[5,26]^

There were several interesting findings in our study. Although our study's primary outcome was focused on the RC, we also analyzed the entire colon. Interestingly, using a transparent cap for colonoscopy significantly lowered the ADR in the TC, even if adjusted by other factors (Table 3). There have been many previous studies to prove the efficacy of the CAC, and the results have been variable.^[13,28–30]^ The most recent randomized control trial was performed in the United States.^[31]^ Most of the patients were Hispanic and CAC improved the advanced ADR (AADR), but not the ADR. We had a similar result, in that CAC improved the AADR, but not the ADR. For ADR, the CAC group was even significantly lower and similar result has been reported in a previous study.^[30]^ This result may be explained by the fact that ADR and AADR are independent colonoscopy quality indicators, first demonstrated in 2013. Greenspan et al^[32]^ said that a high ADR endoscopist can have low AADR, and low ADR endoscopist can have a high AADR. A similar report was published in Korea^[33]^ and similar results were obtained in our study (Tables 2 and 3). Therefore, CAC may still have many controversies. However, CAC may have an effect to prevent ICC because its effect on increasing AADR in the entire colon cannot be ignored. Further studies must proceed to prove the CAC effect in ICCs.

In our study, CWT of the retroflexion group (2.73 ± 0.83 minutes) was significantly longer than that of the nonretroflexion group (2.20 ± 1.08 minutes), as expected from the hypothesis. However, the retroflexion in the RC did not improve the ADR and AADR in the RC, contrary with the findings in previous studies.^[16,17,34]^ The total patient number of the retroflexion group was small and only 3 gastroenterologists performed the retroflexion, so it is hard to compare with previous retroflexion studies. Moreover, the sedative state did not improve ADR. We used midazolam and/or propofol for the sedation, and the sedation had nothing to do with the ADR, concordant with the previous studies.^[14,27,35]^

**Figure 2:**
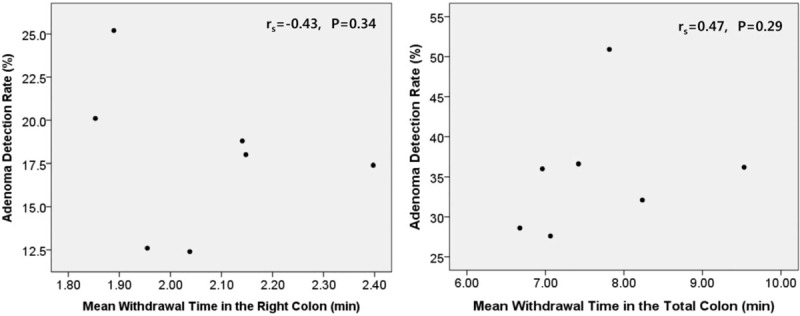
Relationship between mean withdrawal time in colonoscopies where no polyps are removed compared to each gastroenterologists’ adenoma detection rates. There was no significant correlation both in the right and total colon, using Spearman rank-correlation analysis.

Figure 2 shows the mean rate of detection of adenomas by individual endoscopists, plotted against their mean CWTs for procedures in which no polyps were removed. There was no correlation between withdrawal times and the rate of detection of adenomas in the both right and TC. This means that colonoscopic withdrawal techniques are important to increase the adenoma detection.^[36–38]^ Rex showed that ADR depended on 4 quality criteria of withdrawal technique: examining the proximal sides of flexures, folds, and valves, cleaning and suctioning, adequacy of distention, and adequacy of time spent viewing.^[39]^ The withdrawal time belongs to the fourth element. Colonoscopic withdrawal technique is as important as withdrawal time,^[23]^ and we should keep in mind about it.

There are several limitations in our study. First, due to the retrospective design of the study, PACS images were used to calculate the withdrawal time, which could potentially cause bias. However, the OR of withdrawal time for adenoma detection in the RC was clearly higher than 1 (OR 1.89, 95% CI 1.71–2.08, *P* < .001), and a large number of patients enrolled in this study may minimized such bias. The CWT for the entire colon can have such bias, but the ascending colon is short of its length and therefore the potential bias may less likely to occur. As shown in Figure 1, the total image of the RC was <20 cuts, whereas the total image of the entire colon was highest to 150 cuts. Second, the RC defined as cecum to hepatic flexure is maybe too narrow to expect the effect of preventing ICCs. However, ICCs are partly due to missed lesions,^[40,41]^ and the majority of ICCs are found in the proximal colon, especially in the RC, and only a minority in the transverse colon.^[19,42,43]^ The fold of ascending colon is deeper than that of transverse colon and missed lesions more occur in the RC. Based on these premises, the effort to increase ADR in the RC via longer withdrawal time cannot be ignored in contribution to ICC prevention. Tsai et al reported that shorter ascending CWT was associated with ICCs. The mean ascending colon CWT of the ICC group was 2 minutes, whereas the mean ascending colon CWT of the no ICC group was 5 minutes. It is somewhat regrettable that they did not calculate the cut off value of the CWT in the ascending colon when ICC occurred.^[8]^

We tried to calculate the withdrawal time of proximal colon (cecum to splenic flexure). However, because of its retrospective design, it was hard to identify the splenic flexure via PACS, whereas hepatic flexure was easily discernible. Further prospective studies to find the optimum CWT in the proximal colon should be encouraged.

The strength of our study is that this is the first study to directly compare the association between the CWT and ADR of the RC. There was significant association between RC CWT and the RC ADR, suggesting the importance of careful withdrawal in the RC. And our study represented similar result of optimum CWT in the entire colon, making the reliability of the result of optimum CWT in the RC. Second, we adjusted by endoscopists withdrawal technique. As shown in Figure 2, gastroenterologist withdrawal technique was apart from the CWT. Many previous studies did not account for this. However, we adjusted by even endoscopists withdrawal technique in the multivariable analysis (Tables 2 and 3), and CWT was still the major factor of the detection of adenomas and advanced adenomas. Third, the number of patients involved in this study was large enough to guarantee the statistical power of the study. Fourth, because of the retrospective design, the gastroenterologists were unaware of the collection of their procedural data. Such “blinding” enabled the evaluation of colonoscopic technique without the influence of observation. Previous studies, the physicians knew that their procedural information would be collected; possibly influencing endoscopic timing and technique.

In conclusion, longer CWT in the RC was associated with improving ADR and AADR of the RC, and we suggest that the optimum CWT in the RC should exceed 3 minutes. Moreover, longer CWT in the TC was associated with improving ADR and AADR of the TC and, considering the “ceiling effect,” the optimum CWT in the TC should exceed 9 minutes. Although CAC decreased the ADR in the TC, it improved the AADR in the TC. High AADR may contribute more than ADR to prevent ICC and CAC may have a role in preventing ICC, and therefore these factors should be considered.

## Author contributions

**Conceptualization: Sun Hyung Kang.**

**Data curation: Hyuk Soo Eun.**

**Formal analysis: Ju Seok Kim.**

**Investigation: Jong Seok Joo.**

**Methodology: Hee Seok Moon.**

**Project administration: Eaum Seok Lee.**

**Resources: Seok Hyun Kim.**

**Software: Jae Kyu Sung.**

**Supervision: Byung Seok Lee, Hyun Yong Jeong.**

**Writing – original draft: Gee Young Yun.**
